# Virus infection and vesicle trafficking

**DOI:** 10.3389/fimmu.2025.1682139

**Published:** 2025-10-09

**Authors:** Guo-Xiu Cao, Fan-Xin Liu, Chun-Chun Meng, Chan Ding, Jun Dai, Xu-Sheng Qiu

**Affiliations:** ^1^ Guizhou Aerospace Hospital, Dermatolog, Zunyi, China; ^2^ Key Laboratory of Basic Pharmacology of Guizhou Province and School of Pharmacy, Zunyi Medical University, Zunyi, China; ^3^ Shanghai Veterinary Research Institute, Chinese Academy of Agricultural Sciences, Shanghai, China

**Keywords:** vesicle trafficking, virus, hijack, immune escape, antiviral

## Abstract

Vesicle trafficking mechanisms play indispensable roles throughout the viral replication cycle, though their stage-specific regulatory mechanisms during infection require further elucidation. Notably, the latest research reveals that diverse viruses strategically exploit host vesicle trafficking proteins to orchestrate critical infection phases, including receptor-mediated endocytosis initiation, viral attachment/membrane fusion, intracellular component transport, genome replication complex reorganization, and viral assembly/budding. By commandeering these trafficking pathways, viruses not only optimize cellular entry efficiency and immune evasion capabilities but also establish dynamic organelle microenvironments conducive to genome replication. Consequently, therapeutic strategies targeting vesicular transport nodes—through functional inhibition of trafficking proteins or disruption of vesicle homeostasis—have emerged as promising antiviral approaches with clinical translation potential. This review systematically examines viral phase-dependent mechanisms of host vesicular networks, elucidates infection optimization through transport pathway subversion, and evaluates current efforts in developing vesicle-targeted antivirals, thereby providing conceptual frameworks for novel therapeutic design.

## Highlights

- Viruses hijack vesicle trafficking proteins to enhance replication;
- Viruses exploit vesicle trafficking to immune evasion;
- Targeting vesicle system enables multi-pronged antiviral strategies.

## 1 Introduction

Vesicular trafficking facilitates biomolecule transport through intracellular movement, membrane fusion and budding (1), thereby supporting cellular cargo distribution and secretion. This dynamic process is mediated by interconnected compartments—including the endoplasmic reticulum (ER), Golgi apparatus, trans-Golgi network (TGN), endosomes, and lysosomes—which collectively form an integrated cargo-processing network. These compartments operate through five evolutionarily conserved pathways that govern this system: (1) ER-to-Golgi transport (2), (2) Golgi-to-ER recycling (3), (3) TGN sorting (4), (4) endocytic trafficking (5), and (5) autophagic flux (6). These pathways are tightly regulated by key molecular components such as clathrin (7), adaptor protein complexes (8), dynein (9) and Rab proteins (10). Furthermore, emerging evidence highlights non-canonical vesicle trafficking pathways. For example, mitochondria-derived vesicles (MDVs) mediate UFMylation-dependent sorting of MAVS for lysosomal elimination (11), while Golgi-derived secretory granules (SGs) coordinate amphisome-mediated fusion through phosphoinositide conversion cascades (PI(3,4,5)P_3_→PI(4,5)P_2_) and CD63/PTPN9-regulated membrane fission (12). Building on these findings, intriguingly, core machinery like SNAREs and Rabs—originally characterized in canonical pathways—also orchestrate non-canonical trafficking through competitive SNARE complex assembly (e.g., Ykt6-Syx17-Snap29 vs Vamp7-Syx17-Snap29) and structure-specific regulatory mechanisms (RQ mutation tolerance in Ykt6’s zero ionic layer) (13), whereas Rab effectors coordinate vesicle tethering via HOPS complex interactions (14). Crucially, the conservation of both canonical and non-canonical trafficking modules not only underpins their essential role in cellular homeostasis but also creates vulnerabilities exploited by viruses, thereby amplifying the complexity of host-pathogen interdependence.

**Figure 1:**
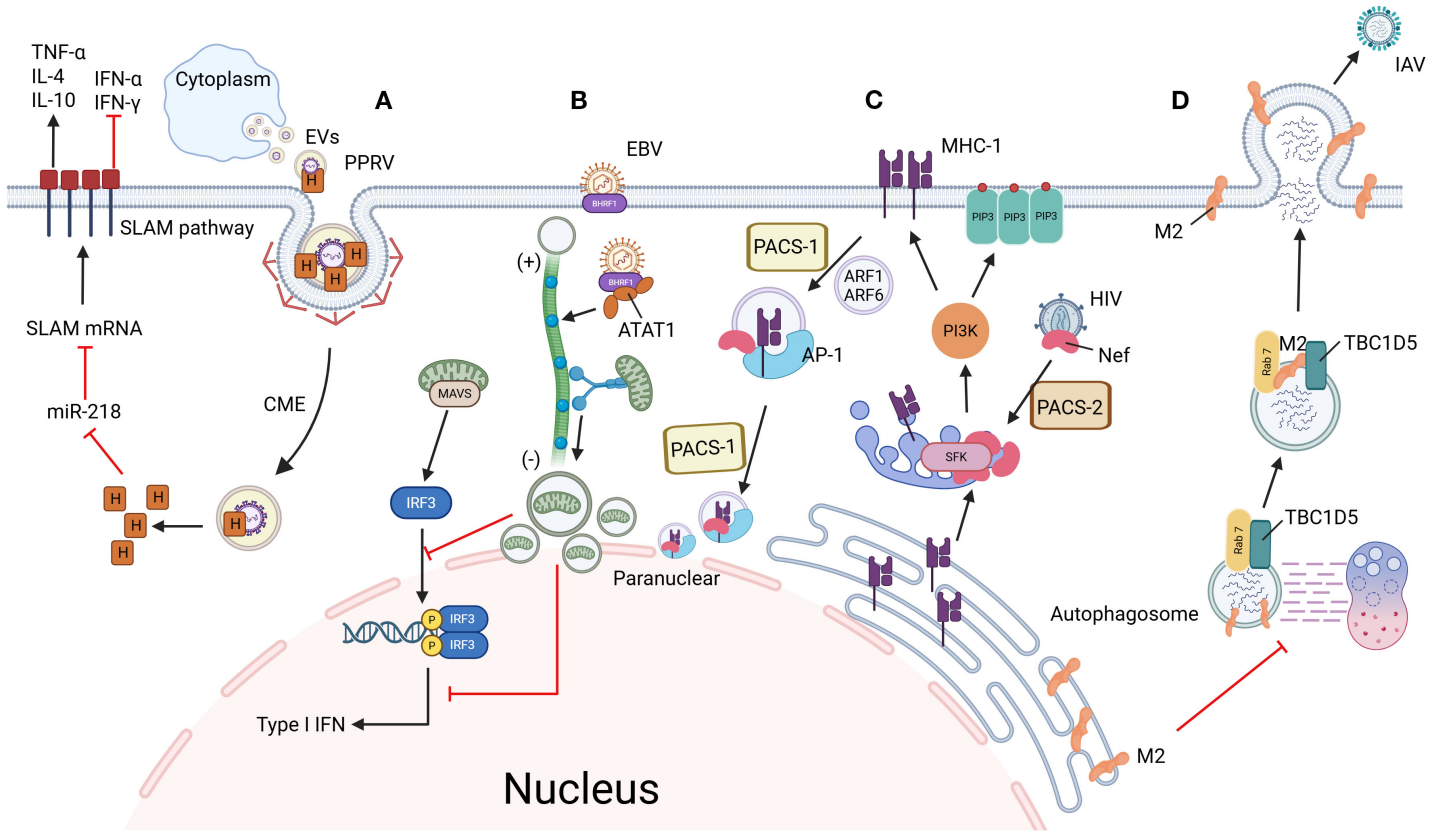
Mechanisms of viral immune evasion through hijacking vesicle trafficking systems. **(A)** PPRV enhances infectivity via SLAM receptor modulation: PPRV enters host cells through CME using extracellular vesicles. Its H protein suppresses miR-218, thereby upregulating SLAM receptor expression to facilitate viral entry. Concurrently, PPRV activates the SLAM signaling pathway, altering cytokine expression to create an immunosuppressive microenvironment and promote persistent infection. **(B)** EBV disrupts mitochondrial dynamics to evade innate immunity: EBV-encoded BHRF1 induces microtubule hyperacetylation, which hijacks dynein-mediated retrograde transport. This drives mitochondrial clustering near the nucleus, disrupting their dynamic network required for MAVS assembly. Consequently, interferon production is blocked, allowing EBV to evade innate immune surveillance. **(C)** HIV hijacks MHC-I recycling via AP-1/PACS-1 axis: HIV Nef is recruited to the TGN by PACS-2, where it activates SFK kinases and triggers PI3K-dependent PIP3 accumulation on the plasma membrane. Activated PI3K recruits ARF1/6 to drive MHC-I internalization into coated vesicles. Internalized MHC-I forms a ternary complex with Nef and AP-1, which is redirected to the perinuclear region via PACS-1-mediated sorting, ultimately blocking MHC-I recycling to the cell surface and suppressing antigen presentation. **(D)** IAV blocks autophagic degradation via Rab7 inactivation: IAV M2 protein disrupts the interaction between Rab7 GTPase and its GAP protein TBC1D5, preventing GTP hydrolysis and locking Rab7 in an inactive state. This failure of Rab7 activation impairs autophagosome-lysosome fusion, allowing viral particles to escape lysosomal degradation. By maintaining intracellular viral survival and facilitating plasma membrane budding, M2 ensures efficient IAV propagation. Figure created with BioRender.

Virus particles are minimally composed of a nucleic acid genome (RNA or DNA) enclosed within a protein capsid, often surrounded by a host-derived lipid envelope studded with viral glycoproteins. Their replication cycle progresses through four host-dependent stages: entry/uncoating (15), genome replication (16), protein synthesis/processing (17), and virion assembly/egress (18). These processes critically exploit the dynamic membrane systems of eukaryotic cells, which perpetually remodel membrane-bound organelles—such as the endoplasmic reticulum, Golgi apparatus, and endosomes—to regulate interorganellar connectivity in response to intracellular and extracellular signals (19). Following cellular entry, viruses strategically hijack vesicular trafficking pathways to establish replication hubs. For example, (+) ssRNA viruses like SARS-CoV-2 co-opt endoplasmic reticulum-derived vesicles for replication organelle formation (20), while enveloped viruses such as HIV-1 redirect Rab GTPase-regulated endosomal networks for virion budding (21). Such viral interference restructures organelle networks to optimize resource allocation for replication (22) or repurposes transport vesicles for immune evasion and dissemination (23). These adaptations highlight how pathogens exploit conserved membrane remodeling mechanisms to balance replication efficiency with stealth, often at the cost of host cell homeostasis (**Figure 1**).

Notwithstanding the pivotal role of vesicular trafficking across viral infection stages, its specific contribution to viral replication remains to be determined. Based on the close relationship between vesicle trafficking related proteins and virus replication, this review systematically summarizes the functional characterization of key trafficking proteins hijacked during infection, comparative analysis of molecular mechanisms through which diverse viruses subvert vesicular pathways, and deeply analyzes how these mechanisms cooperate to promote the dynamic process of virus replication and transmission.

## 2 Virus infection and vesicular trafficking-related host proteins

Vesicular trafficking mediates viral replication through host factor cooperativity, with mechanistic studies revealing lineage-specific adaptations. Strikingly, COPII vesicles drive rotavirus particle maturation through direct interaction with the enterotoxin NSP4 (24), while COPI components scaffold replication complex assembly during Drosophila C virus infection (25). Parallel analyses of divergent systems reveal distinct host machinery dependencies: HCV cellular entry requires Septin-6 isoforms for membrane anchoring (26), whereas DENV replication in mosquito cells hinges on Septin-2-mediated modulation of ER rearrangement (27). To systematically dissect these interactions, the following subsections focus on key vesicle trafficking components—clathrin-coated vesicles, dynein motor complexes, adaptor protein assemblies, and Rab GTPase networks—that enable viral exploitation of host transport systems. These evidences suggest that the virus has established a conserved evolutionary strategy to promote its own replication by hijacking the vesicle associated protein induced transport system (28) (**Table 1**).

**Table 1:** Viral replication is closely related to vesicle trafficking related proteins.

Virus		Rab proteins									Clathrin	Dynein	Adaptor protein complexes			
		rab1	rab5	rab6	rab7	rab8	rab9	rab11	rab18	rab14			AP-1	AP-2	AP-3	AP-4
Adenoviridae	EDSV										(256)					
Arenaviridae	LASV										(38)					
	LCMV				(257)						(258)					(259)
Arteriviridae	PRRSV		(260)		(260)			(261)	(172)		(35)					
Asfarviridae	ASFV	(262)	(263)		(263)						(264)	(265)	(121)			
Coronaviridae	PEDV				(266)			(267)			(268)	(103)				
	PHEV		(269)		(269)		(269)				(269)	(269)				
	SARS-CoV-2	(10)	(270)		(271)			(272)		(273)	(44)	(274)		(275)	(155)	
Euroniviridae	YHV		(276)		(277)			(278)			(279)			(279)		
Filoviridae	EBOV				(280)						(281)	(282)				
	MARV							(174)								
Flaviviridae	CSFV	(283)	(51)		(97)	(284)	(54)	(53)	(285)	(286)	(51)	(97)				
	DENV		(287)		(287)			(287)	(171)		(36)	(288)	(123)	(141)		
	WNV	(289)	(290)		(291)	(292)					(293)					
	ZIKV	(294)	(295)		(296)						(297)	(298)				
Hepadnavividae	HBV		(299)		(299)		(300)	(301)			(302)	(303)		(139)		
	HCV	(182)	(304)	(304)	(304)		(184)	(304)	(180)		(305)	(306)	(111)	(111)		(111)
	HDV										(307)					
	HEV		(308)		(308)			(309)			(310)	(311)	(105)			
Herpesviridae	EHV-1										(312)	(313)				
	HSV-1	(186)	(185)	(188)	(314)		(187)	(185)			(62)	(315)	(124)			
	PRV			(173)							(140)	(316)	(122)	(317)		
Nairoviridae	CCHFV		(318)								(319)			(320)		
Nimaviridae	WSSV	(321)	(322)	(323)	(324)		(325)	(326)			(327)	(328)		(329)		
Paramyxoviridae	NDV		(330)								(331)					
	RSV		(332)		(14)			(175)	(333)		(334)	(335)			(150)	
Parvoviridae	CPV		(336)		(336)			(336)			(337)	(338)				
Picornaviridae	HAV										(339)					
	HPEV-1										(340)					
Pneumoviridae	HRSV														(153)	
Retroviridae	HIV-1	(341)	(342)	(343)	(342)			(344)		(345)	(34)	(100)	(109)	(135)	(148)	(346)
	HIV-2										(347)			(133)	(150)	
Rhabdoviridae	RABV		(348)		(348)						(349)	(350)		(351)		
Togaviridae	CHIKV		(352)								(353)					

### 2.1 Clathrin

The molecular mechanism of virus entry into host cells is the key to regulate infectivity, and clathrin mediated endocytosis (CME) has been established as the main entry pathway after long-term research. During CME, clathrin trimers composed of heavy and light chains self-assemble to form a polyhedral lattice, driving the generation of clathrin coated pits (CCP) (29, 30). This mechanism confers dual virological advantages: hijacking CME not only enhances invasion efficiency but also promotes rapid entry into endosomal compartments via clathrin-encapsulated vesicles. Strategically, this pathway evades cell membrane receptor surveillance while simultaneously delaying activation of host innate immune responses, thereby extending the temporal window for successful infection establishment (31).

As the central regulatory framework of mammalian vesicular transport, CME orchestrates spatiotemporal coordination of diverse cargoes—including signaling molecules, synaptic vesicles, and surface receptors—through clathrin-coated vesicle formation (32, 33). This hub-like regulatory capacity establishes CME as the preferred entry portal for multiple enveloped viruses, with canonical examples encompassing HIV-1 (34), PPRSV (35) and DENV (36). Notably, the evolutionary co-option of CME by viruses underscores the intricate equilibrium in host-pathogen dynamics: while utilizing this highly conserved pathway maximizes viral entry efficiency, it concurrently preserves host countermeasures targeting critical CME nodes. In HIV-1 pathogenesis, post-CME entry triggers a dual immunosuppressive cascade—accelerated endosomal acidification facilitates capsid disassembly while synchronized viral protease activation degrades restriction factors (37). This dual strategy of physical compartmentalization and enzymatic elimination substantially diminishes host antiviral capacity. Crucially, CME’s systemic influence spans the entire viral life cycle, governing initial receptor engagement (38), cellular invasion (39, 40), intracellular trafficking (41) and progeny virion egress (42, 43), thereby demonstrating its critical pathological significance as a fundamental cellular process co-opted by viral pathogens.

**Figure 2:**
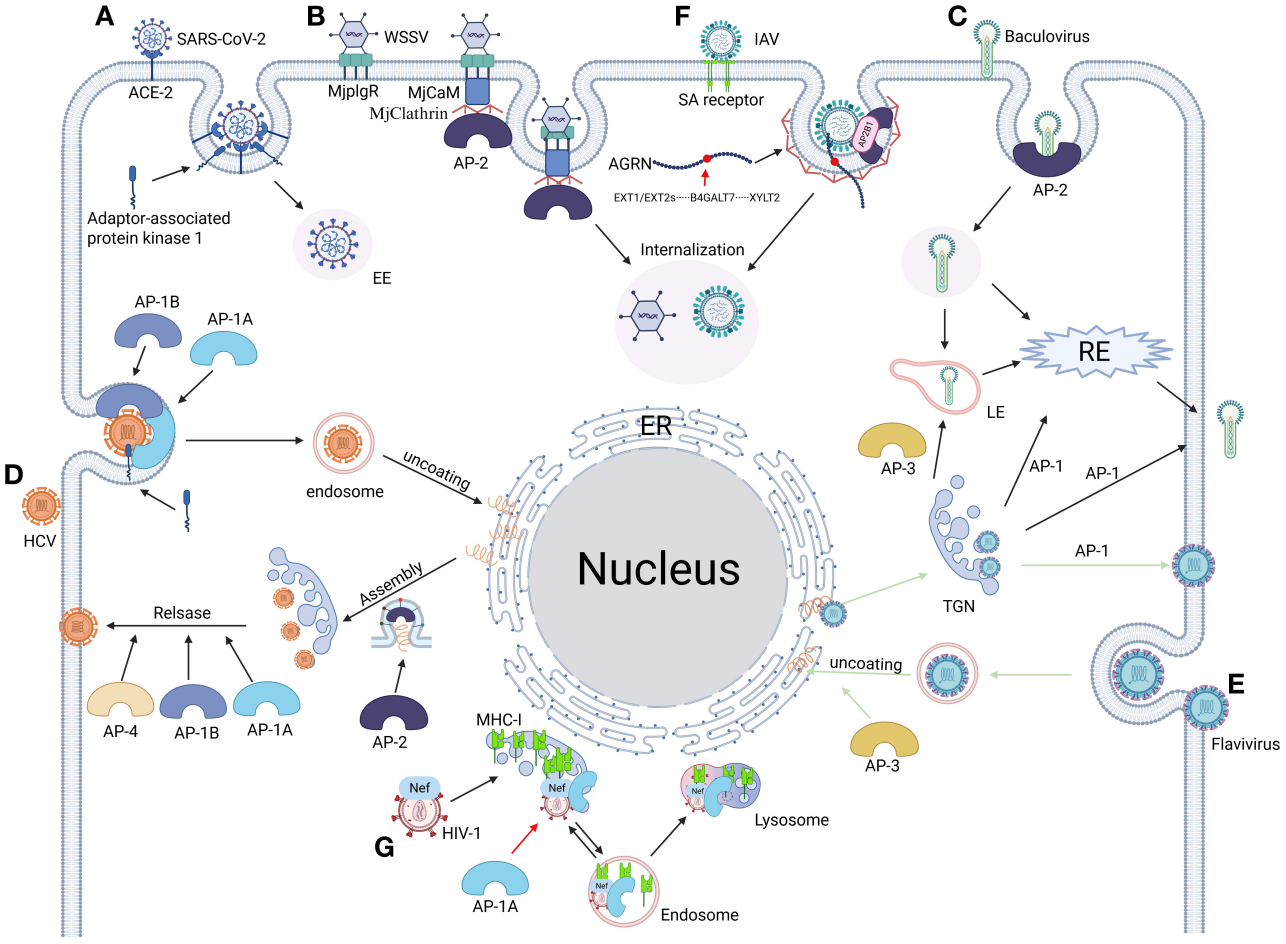
From invasion to release: the full cycle of viral replication mediated by adaptor proteins. **(A)** SARS-CoV-2 hijacks clathrin regulator AAK1 (AP-2-associated kinase 1) to drive AP-2-dependent endocytosis, mediating efficient viral entry (155, 275). **(B)** WSSV engages host receptor MjPlgR through luminal domain binding, activating a stepwise assembly: the receptor’s cytosolic domain recruits calmodulin (MjCaM), propagates a CaM-clathrin-AP2 cascade, and executes clathrin-mediated viral uptake (329). **(C)** Baculovirus coopts AP complexes for compartment-specific trafficking: AP-1 orchestrates virion assembly at the TGN and routes progeny to RE or plasma membranes. AP-2 steers clathrin-mediated viral internalization into EE and subsequent RE trafficking. AP-3 mediates the formation and transport of virions from the Golgi membrane to LE (410). **(D)** HCV commandeers adaptor protein complexes AP-1a, AP-1b, AP-2, and AP-4 to orchestrate stage-specific vesicular trafficking: AP-1a/1b-AP-2 axis directs entry, assembly, and cell-cell spread via host/viral protein interfaces. AP-1a/1b-AP-4 nexus coordinates late egress and cell-free virion dissemination (111). **(E)** Flaviviruses commandeer AP complexes for compartmentalized infection: AP-3 licenses endosomal maturation, enabling capsid uncoating in LE for cytoplasmic RNA release. AP-1 orchestrates TGN egress by packaging immature virions into secretory vesicles after trans-Golgi trafficking (151). **(F)** IAV undergoes heparan sulfate (HS)-mediated transport to the plasma membrane via Agrn protein binding, interacts with its HA surface protein, and recruits the AP2B1 subunit of the endocytic adaptor complex AP-2. This step initiates AP-2-dependent endocytosis, thereby achieving viral internalization (411). **(G)** The HIV-1 Nef protein subverts host immunity by hijacking the AP-1 complex’s μ1 subunit, redirecting newly synthesized MHC-I molecules from the Golgi apparatus to lysosomal degradation. This immune evasion strategy effectively blocks antigen presentation through Nef-mediated bridging of AP-1 to a non-canonical binding motif on the cytoplasmic tail of MHC-I (412). Figure created with BioRender.

**Figure 3:**
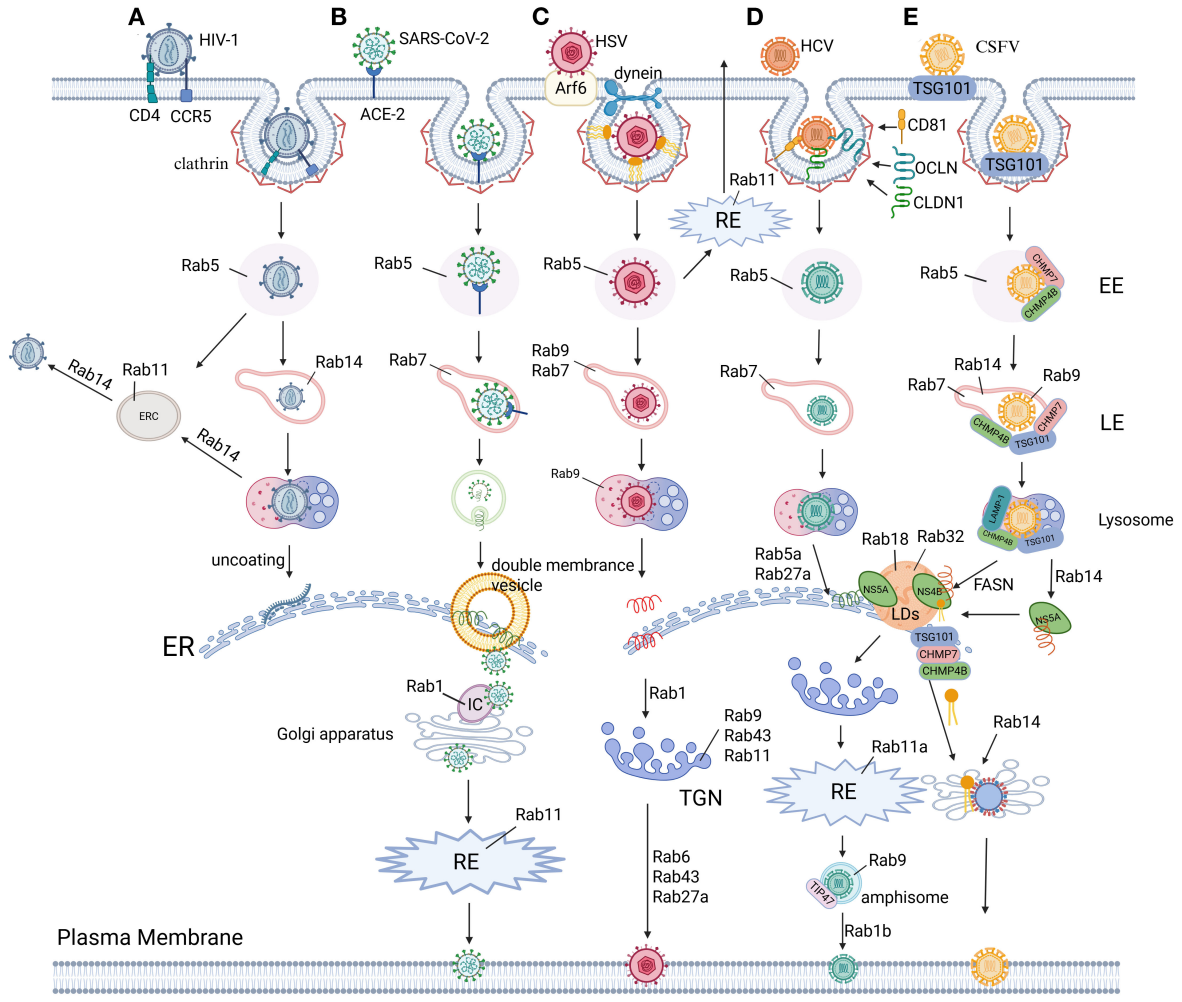
The role of Rab proteins in virus replication. **(A)** HIV-1 Env hijacks Rab GTPases for compartmentalized trafficking: Rab5 licenses EE sorting post-internalization. RAB14-dependent triage partitions Env into Rab11-ERC or LE/lysosomal degradation via endocytic intermediates. In addition, RAB14 steers retrograde trafficking of intact Env to the ER for viral replication. RAB14 restricts Env’s secretory axis, limiting PM delivery of viral glycoproteins (342, 344, 345). **(B)** SARS-CoV-2 entry initiates via ACE2 receptor binding and EMC. Post-internalization, the nucleocapsid uncoats in the cytoplasm to release the viral RNA genome. Viral envelopment occurs during ER-Golgi intermediate compartment (ERGIC) budding, followed by Rab1- and Rab11-dependent vesicular trafficking to the plasma membrane for virion release (10, 270–272). **(C)** HSV hijacks Rab GTPase networks for compartmentalized trafficking: CME via Rab5 delivers virions to EE, followed by Rab7/Rab9-mediated sorting into LE/lysosomes enroute to the ER for replication. Rab1 coordinates ER-to-Golgi transport of envelope proteins, while Rab43/Rab9/Rab11 mediate endosome-to-TGN trafficking for secondary envelopment. Export via trans-Golgi secretory vesicles requires Rab6, Rab43, and Rab27A, with Rab11 alternatively recycling plasma membrane-bound virions through RE (185–188, 314). **(D)** HCV hijacks Rab GTPases for compartmentalized trafficking: Post-attachment to CD81/CLDN1/OCLN, clathrin-mediated entry delivers virions to Rab5/Rab7-regulated endolysosomal transport for uncoating. Replication at the ER is coordinated by RAB5A and Rab27A, while Rab18/Rab32 direct core/NS5A trafficking to assembly membranes. Secretion involves Rab1b-mediated Golgi targeting, Rab11a-RE routing, and Rab9/TIP47-dependent exocytic sorting for non-lytic egress (180, 182, 184, 304). **(E)** CSFV hijacks ESCRT machinery and Rab GTPases for compartmentalized replication: Clathrin/TSG101-mediated entry delivers virions to Rab5-positive EE via ESCRT-I/III (TSG101/VPS25/CHMP4b/7). Rab5/Rab7/9/14 coordinate endolysosomal trafficking, with lysosomal LAMP-1/TSG101/CHMP4b enabling uncoating and ER-directed RNA release. NS4B recruits FASN to ER-lipid droplet interfaces, amplifying fatty acid synthesis via Rab18-FASN feedback to boost replication. Concurrently, NS5A redirects RAB14 to ER-Golgi ceramide flux, fueling sphingomyelin-enriched membranes for replication. Immature virions assemble in the ER lumen, maturing in the Golgi via Rab-dependent secretory trafficking (51, 53, 54, 97, 283–286). Figure created with BioRender.

CME serves as a fundamental entry strategy for diverse enveloped viruses, though viral molecular mechanisms exhibit marked heterogeneity in CCP dynamics and immune modulation. While SARS-CoV-2 (44) (**Figures 2A**, **3B**), IAV, and PEDV all exploit CME for invasion, they employ distinct strategies: SARS-CoV-2 and IAV activate canonical CME by recruiting the AP-2 complex for Rab5-dependent trafficking to early endosomes (EE), where pH-dependent fusion occurs. Notably, SARS-CoV-2 selectively retains clathrin adaptors to suppress STING-dependent immune signaling during endosomal transport, enabling early immune evasion (45). In contrast, PEDV employs an evolved RNASEK-EPS15 hijacking mechanism—its S2 glycoprotein binds RNASEK (a CME initiation factor) to drive EPS15/AP-2/clathrin assembly at CCPs. This facilitates transport to late endosomes (LE) for genome release while boosting virion-endosome affinity and accelerating clathrin polymerization (46–48). Concurrently, this pathway redirects virions to immune-silent compartments and attenuates adaptive immunity by blocking MHC-I antigen presentation (49). Analogously, Peste des petits ruminants virus (PPRV) utilizes CME through extracellular vesicles but enhances infectivity via SLAM receptor modulation: its H protein suppresses miR-218 to upregulate SLAM expression, while activating SLAM signaling to reshape cytokine expression and establish an immunosuppressive microenvironment for persistent infection (50) (**Figure 1A**). Collectively, these divergent mechanisms underscore the dual role of the clathrin system as both a conserved entry gateway and a pliable interface for viral adaptation through tailored CCP regulation and immune evasion tactics.

In addition to the aforementioned viruses, the infection cycles of CSFV and ZIKV are also intricately linked to CME mechanisms. CSFV utilizes clathrin-coated vesicles to traffic into endolysosomal compartments (51) (**Figure 3E**), where its NS3 protease specifically cleaves mitochondrial antiviral-signaling protein (MAVS) to block type I interferon (IFN) signaling (52), thereby facilitating subsequent ER membrane remodeling and replication complex assembly (6, 53, 54). Similarly, ZIKV exploits HAVCR1-mediated clathrin endocytosis for endosomal entry (55), with its uncoating and replication processes being coordinately regulated by ubiquitination modifications (56, 57) and endoplasmic reticulum membrane reorganization (58–60). Strikingly, recent studies demonstrate that ZIKV’s NS4A protein hijacks the CME transport pathway to translocate to mitochondria, where it binds MAVS to inhibit RIG-I signaling complex formation (61). This vesicle-coupled immune evasion strategy markedly enhances viral replication efficiency. Collectively, these examples underscore CME’s conserved biological role across phylogenetically distinct viruses: by precisely modulating endosomal trafficking and immune signaling checkpoints, it establishes a molecular foundation for viruses to gain early infection advantages.

While CME persists as the canonical entry pathway for numerous viruses, emerging evidence delineates alternative mechanisms that expand our understanding of viral entry biology. Recent studies have characterized non-canonical strategies: HSV-1 hijacks PAK1-dependent macropinocytosis through Na^+^/H^+^ exchanger activation (62, 63), while LASV exploits cholesterol-rich membrane microdomains via glycoprotein-mediated docking before leveraging Rab7-coupled endosomal maturation for fusion (38, 64). These discoveries not only reaffirm CME’s central role in viral pathogenesis but also reveal the increasing complexity of entry route diversification, demanding refined models of tropism to inform therapeutic targeting. Crucially, such alternative pathways exhibit intrinsic immunomodulatory properties—HSV-1’s macropinocytic entry shortens cytoplasmic exposure of viral nucleic acids to limit pattern recognition receptor activation (63), whereas LASV’s preferential use of LE as fusion sites circumvents TLR3-mediated surveillance mechanisms predominant in early compartments (65). Collectively, these findings illustrate the dual evolutionary logic of viral entry strategies: they balance mechanistic conservation (leveraging established pathways like CME) with context-driven innovation (adapting alternative routes), ultimately optimizing host-cell adaptation while evading immune detection. This dynamic equilibrium positions viral entry as a prime target for therapies exploiting pathway vulnerabilities at the host-pathogen interface.

### 2.2 Dynein

Dynein, a microtubule-associated motor protein, coordinates intracellular trafficking of cellular cargo and viral particles across critical stages of pathogenesis. This molecular motor directly facilitates viral entry (66), genome replication (14), virion assembly (67), and progeny release (68), with its transport machinery hijacked by viruses to propel capsids through the cytoplasmic milieu (69). Notably, dynein-mediated microtubule trafficking exhibits a precisely balanced dual role in immune regulation during infection. In host defense, dynein collaborates with TAOK1 kinase to translocate TAOK1 along microtubules to the perinuclear region, where phosphorylation-dependent binding to IRF3 stabilizes the TBK1-IRF3 signaling complex. This spatial reorganization activates the type I interferon pathway, inducing antiviral effector protein expression to establish a cell-wide antiviral state (70). Conversely, viruses exploit dynein-mediated pathways to subvert immunity through distinct mechanisms. For instance, IAV interacts with dynein via the LC3-PCNT autophagy adaptor complex, which simultaneously promotes nucleocapsid uncoating and disrupts antiviral signal translocation to perinuclear reaction centers. Mechanistically, after endosomal fusion releases vRNPs into the cytoplasm, the LC3-PCNT complex acts as a dynein adaptor through two distinct interactions: (1) LC3 binds to vRNP-associated M1 matrix proteins, while (2) PCNT recruits dynein heavy chains. This assembly enables dynein-mediated transport of vRNPs along microtubules, facilitating their uncoating and nuclear import (71). Concurrently, viral nucleoprotein (NP) hijacks mitophagy to degrade mitochondrial antiviral-signaling protein (MAVS), thereby blocking the assembly of perinuclear antiviral complexes dependent on MAVS-mediated RIG-I signaling (72). By spatiotemporally decoupling host immune responses from viral replication, this strategy enables immune escape and infection spread. Sizmilarly, EBV employs an alternative strategy through its BHRF1 protein, which induces microtubule hyperacetylation to hijack dynein-mediated retrograde transport. This drives mitochondrial clustering near the nucleus, disrupting the dynamic mitochondrial network required for MAVS assembly. Consequently, interferon production is blocked, allowing EBV to evade innate immune surveillance (73) (**Figure 1B**). These contrasting mechanisms underscore dynein’s dual nature: while essential for antiviral signaling, its trafficking pathways are co-opted by viruses to either spatially coordinate immune suppression or dismantle organelle-based antiviral platforms, highlighting its pivotal yet conflicted role in infection outcomes.

**Figure 4:**
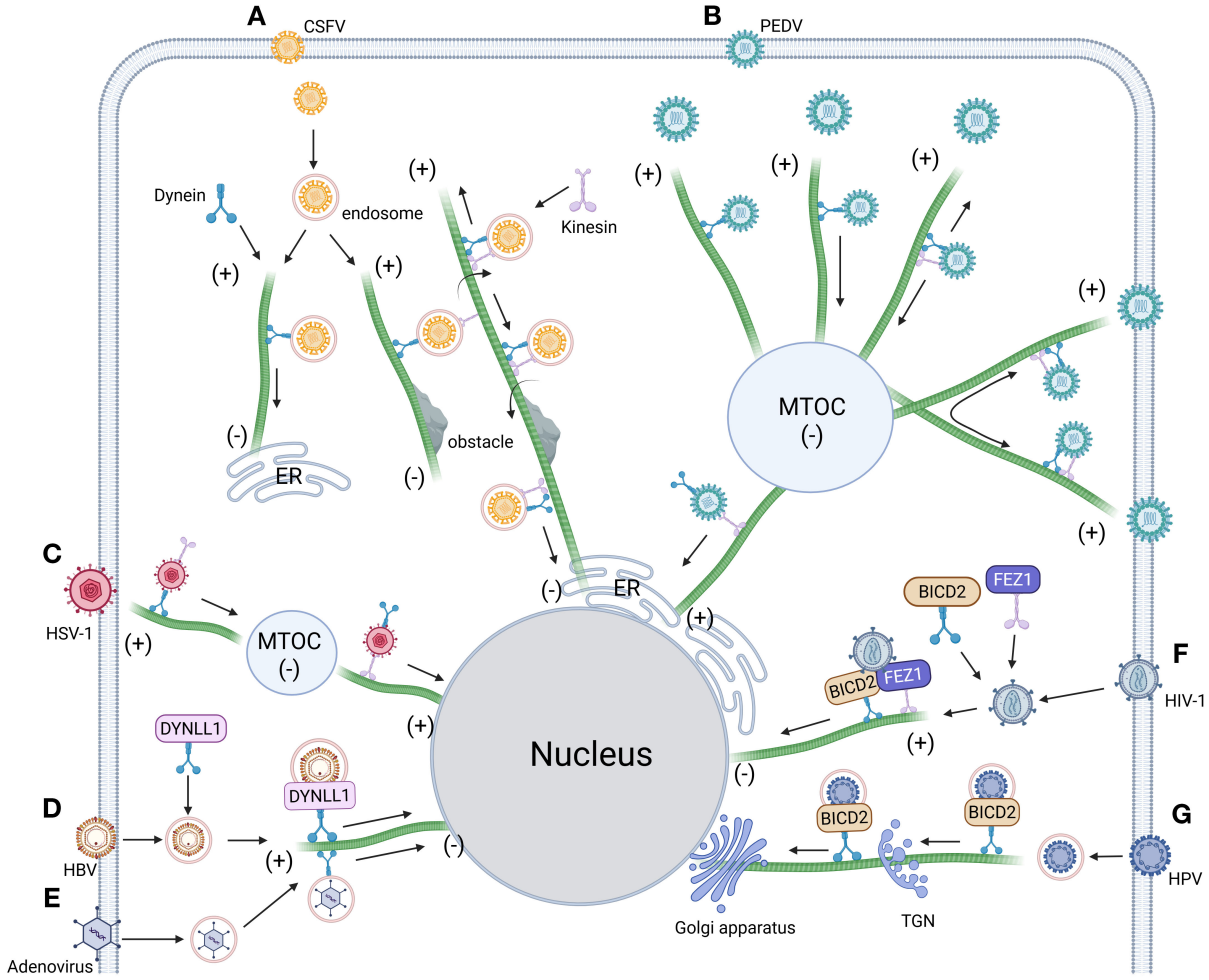
Intracellular trafficking model of virus dependent dynein. **(A)** Dynein orchestrates CSFV-endosome transport along microtubules toward polarized regions. Obstacle encounters trigger dynein-kinesin-1 coordinated navigation for barrier circumvention, followed by resumed microtubule transport culminating in ER docking (97). **(B)** The intercellular transport of PEDV along microtubules driven by dynein and kinesin-1 includes quiescent state, unidirectional movement toward the negative end of microtubules, bidirectional movement along the same microtubule and bidirectional movement along different microtubules (103). **(C)** HSV-1 hijacks the microtubule network to drive retrograde transport of viral cargoes, navigating first to the microtubule organizing center (MTOC) before ultimately localizing to the nuclear compartment (315). **(D)** HBV co-opts dynein light chain DYNLL1 to form capsid-associated complexes, orchestrating microtubule-dependent trafficking toward the nuclear compartment (303). **(E)** Post-entry adenovirus hijacks dynamin-mediated transport to navigate microtubule minus-end-directed trafficking toward the nuclear periphery (408). **(F)** Post-entry HIV-1 co-opts dynein adaptor BICD2 and kinesin-1 adaptor FEZ1 to coordinate microtubule-dependent retrograde transport, ensuring targeted nuclear entry (100). **(G)** HPV commandeers dynein adaptor BICD2 via capsid binding, coupling virions to retrograde motors for microtubule-driven trafficking along the endosome-TGN/Golgi axis (409). Figure created with BioRender.

Structurally, dynein motor family comprises three evolutionarily conserved subfamilies with distinct roles: cytoplasmic dynein-1 (DYN1), responsible for organelle transport (endosomes (74), vesicles (75), lysosomes (76)) and mitotic spindle organization (77); cytoplasmic dynein-2 (DYN2), specialized in retrograde intraflagellar transport (IFT) essential for ciliogenesis (78, 79); and axonemal dynein, which powers motile cilia function (80). Despite sharing structural components—heavy, intermediate, light intermediate, and light chains (81) —DYN1 and DYN2 exhibit functional divergence (82, 83), largely governed by IC-LC subunit interactions that regulate ciliary assembly and retrograde trafficking (84–87). Virions exploit this machinery by hijacking dynein heavy chains to traffic replication complexes to perinuclear regions thereby suppressing type I interferon production (88) (**Figure 4**). Pathologically, dynein-2 subunit mutations correlate with ciliopathies, including thoracic dystrophy (89), ciliary dysfunction (90), and neural tube defects (91). A striking example of dynein’s duality is the light chain Tctex-1, which enhances poliovirus replication by stabilizing host-cell receptor interactions (92) yet is indispensable for DENV assembly, evidenced by defective subviral particle formation in Tctex-1-depleted systems (93). Together, these mechanisms underscore dynein’s central role as a spatiotemporal conductor of viral pathogenesis, coordinating infection dynamics from cellular entry to immune evasion.

Microtubules, polarized cytoskeletal highways composed of α/β-tubulin heterodimers, serve as directional tracks for dynein-driven retrograde transport—a system systematically exploited by viruses to navigate the cytoplasm toward replication sites. These dynamic filaments, operating alongside actin and intermediate filaments in eukaryotic cells (94–96), are co-opted by CSFV following clathrin-mediated entry (51). Live-cell imaging reveals that dynein and kinesin-1 coordinately mediate CSFV trafficking along acetylated microtubules, with their opposing motor activities fine-tuning viral replication efficiency through bidirectional transport regulation (97). After TSG101/Rab7-dependent sorting into LE, CSFV undergoes dynein-powered retrograde transport to lysosomes for capsid disassembly before ER docking (**Figures 4A**, **3E**). Intriguingly, pharmacological inhibition of retrograde motility triggers an adaptive response: kinesin-1-dependent anterograde movement bypasses endolysosomal processing bottlenecks, rerouting virions directly to ER replication hubs. This compensatory mechanism underscores the evolutionary sophistication of viral trafficking strategies in overcoming host transport barriers.

Viral exploitation of host microtubule transport systems reflects an evolutionary balancing act, where pathogens optimize transport efficiency while evading host defense pathways. HIV-1 and HSV-1 exemplify this dichotomy: while both hijack dynein-dependent microtubule transport and utilize dynactin subunit 1 (DCTN1) as a trafficking adaptor (98, 99) (**Figures 4C, F**), their mechanisms diverge significantly. HSV-1 maintains infectivity across varying DCTN1 expression levels without perturbing mitochondrial function, whereas DCTN1 competitively disrupts HIV-1’s early infection cycle by sequestering cytoplasmic linker protein 170 (CLIP-170). CLIP-170 promotes HIV-1 core uncoating and microtubule-dependent nuclear trafficking via its zinc finger 2 (ZnF2) domain, either through direct core binding or recruitment of proviral cofactors (100–102). DCTN1 antagonizes this process by displacing CLIP-170 from viral complexes through competitive ZnF2 domain binding, thereby impairing nuclear transport. These interactions underscore the precise regulatory interplay between microtubule motors (dynein/kinesin) and adaptor proteins in establishing bidirectional transport highways critical for viral pathogenesis. Expanding this paradigm, Hou et al. (103) (**Figure 4B**) identified five microtubule trafficking modes during PEDV infection: anterograde, retrograde, bidirectional oscillation, microtubule-switching, and static anchoring—all mediated by dynein-kinesin-1 coordination. Notably, during static anchoring, PEDV induces microtubule deacetylation to suppress HDAC6-dependent innate immune signaling, creating an immune-evasive niche favorable for replication (104). Collectively, these studies illuminate conserved viral strategies for co-opting microtubule dynamics—orchestrating transport efficiency while neutralizing host defenses through spatiotemporal regulation of motor complexes and post-translational modifications.

### 2.3 Adaptor protein complex

The adaptor protein (AP) complex, a heterotetrameric assembly regulating CME and retroviral morphogenesis (105, 106), comprises five evolutionarily conserved subfamilies (AP-1 to AP-5) that coordinate compartment-specific cargo sorting. These molecular orchestrators exhibit distinct subcellular localizations across membranous organelles (**Figure 2**), governing processes from synaptic vesicle recycling to lysosomal maturation. Mechanistically, AP-2 enhances IAV and coronavirus entry by stabilizing CCP formation (107, 108), while AP-1 facilitates HIV-1 replication by mediating Gag polyprotein trafficking between the TGN and endosomes (109). In contrast, AP-3 supports herpesvirus assembly through lysosome-related organelle (LRO) membrane remodeling (110), whereas AP-4 directs HCV secretion via non-canonical endosomal trafficking pathways that bypass Golgi processing (111). Notably, AP-5 exacerbates ZIKV neurotropism by disrupting autophagosome-lysosome fusion (112), with pathogenic mutations in its associated spastic paraplegia genes (SPG11/SPG15) potentially sensitizing neurons to viral triggers of Parkinsonian neurodegeneration (113). Collectively, these findings position AP complexes as central players in viral pathogenesis, mediating tropism-specific interactions that bridge host trafficking machinery to viral replication cycles.

AP-1, a Golgi basal body/endosome-associated adaptor protein complex, serves as a central hub for intracellular membrane trafficking, with its modular subunit architecture and dynamic functionality making it a strategic focal point in the virus-host interplay (114–116) (**Figure 2D**). This complex exhibits a dual role during viral pathogenesis: its γ subunit-mediated transport machinery supports viral assembly, such as HIV-1 leveraging γ1/γ2 isoforms to antagonize CD317/tetherin and facilitate viral budding (109, 117, 118), while structural vulnerabilities like the μ subunit’s tyrosine-binding domain are hijacked for immune evasion—exemplified by HIV Nef degrading MHC-I via the μ1 subunit (119) and CMV-m154 disrupting AP-1 function to eliminate T cell costimulatory molecules (120). This pan-viral dependency extends across diverse pathogens: ASFV remodels viral factories through CD2v-AP-1 interactions (121), alphaherpesviruses exploit the μ1B subunit to redirect basolateral glycoproteins for transmission tunnel formation (122), DENV and VZV rely on AP-1 for viral factory reorganization and maturation (123, 124), while HEV and HCV employ AP-1A/B isoforms to mediate capsid generation and dual-release pathways, respectively (105). Notably, the functional specialization of AP-1 subtypes—AP-1A orchestrating lysosomal trafficking and AP-1B regulating basolateral recycling (125)—originally evolved as a host defense strategy, yet is subverted by viruses for spatiotemporally targeted attacks. For instance, HIV hijacks the AP-1/PACS-1 axis to manipulate MHC-I recycling: Nef is recruited to the Golgi via PACS-2, activating the SFK-PI3K signaling axis to induce PIP3 accumulation on the plasma membrane. This drives ARF1/6-dependent internalization of MHC-I into AP-1-coated vesicles, which are then redirected by the Nef-AP-1-PACS-1 ternary complex to perinuclear compartments, systematically blocking antigen presentation (126, 127) (**Figure 1C**). These mechanisms underscore AP-1’s structural vulnerabilities—its tyrosine-binding domains and basic clusters—as dual-purpose hubs, essential for physiological trafficking yet exploited as viral targets (128, 129). Consequently, therapeutic strategies targeting AP-1-virus interaction nodes, such as blocking μ1-mediated immune evasion, modulating AAK1/GAK kinase activity, or developing subtype-specific inhibitors, offer precision interventions to disrupt viral lifecycles while preserving cellular homeostasis, thereby opening new avenues for antiviral therapeutics.

The AP-2 complex—a heterotetramer of α, β2, μ2 (AP2M1), and σ2 subunits—acts as a central nexus for viral exploitation of host trafficking machinery, leveraging its structural plasticity and cargo-sorting motifs to mediate infection stages from entry to egress (130–132) (**Figure 2B**). Viral pathogens strategically target AP-2 subunits through precise motif recognition: HIV-2 Env binds the AP-2 complex via its cytoplasmic GYxxΦ motif to orchestrate viral particle release (133), while MLV glycoGag enhances HIV-1 infectivity by engaging the μ2 subunit’s YXXL sorting signal (134). Immune evasion mechanisms are similarly dependent on AP-2, exemplified by HIV-1 Nef hijacking the α/σ2 subunits—via its central loop and critical residues like Arg-134—to drive CD4 receptor endocytosis and immune evasion (135–137). During viral entry, AP-2-dependent CME is exploited by diverse pathogens including HCV (138), HBV (139), PRV (140), and human coronavirus HCoV-229E (107). HBV further underscores this dependency, with its pre-S1 domain directly binding AP-2 and clathrin heavy chain, and pharmacological inhibitors like chlorpromazine blocking infection by disrupting AP-2 function (139). Beyond entry, AP-2 coordinates viral dissemination through subunit-specific mechanisms: DENV requires BIKE kinase-mediated phosphorylation of AP-2 μ2 at T156 to drive both post-internalization trafficking and assembly/egress, with BIKE inhibitors (e.g., 5Z-7-oxozeaenol) potently blocking infection *in vitro* and ex vivo. The antiviral effect is mechanistically linked to BIKE activity, as its overexpression rescues viral replication. Notably, 5Z-7-oxozeaenol exhibits broad-spectrum activity against diverse RNA viruses with a high resistance barrier, highlighting host-targeted antiviral advantages (141, 142). Vaccinia virus recruits AP-2 and clathrin via the NPF motif of its A36 protein to activate Cdc42/N-WASP-driven actin polymerization, enabling cell-to-cell spread (143); and lentiviruses co-opt AP-2 to degrade the host restriction factor tetherin, illustrating dual-pathway hijacking for viral release and immune suppression (144). These findings position BIKE-AP-2 interplay as a nexus linking post-translational modification (T156 phosphorylation) and antiviral gene regulation—e.g., interferon-induced AP2M1 downregulation counteracts viral egress, while DENV subverts this through translational reprogramming (142). These collective interactions position AP-2 as a master regulator of viral pathogenesis, with therapeutic targeting of its subunit interfaces (e.g., μ2 cargo-binding domains), regulatory kinases (AAK1/BIKE), or motif-driven trafficking networks offering a promising strategy for broad-spectrum antivirals that disrupt viral commandeering of host transport systems.

The AP-3 complex, composed of β3a, δ, μ3, and σ3 subunits, orchestrates lysosomal trafficking and organelle biogenesis through δ/μ3 HEAT domain-mediated sorting signal recognition (145, 146), a process co-opted by diverse viruses for pathogenesis. HIV-1 exemplifies multi-layered exploitation: its Gag protein hijacks the δ subunit to redirect virions to late endosomal compartments (147, 148), while μ3b subunit interactions facilitate endosomal membrane penetration during assembly—mechanisms pharmacologically disruptible via targeted inhibition (149, 150). Flaviviruses paradoxically rely on AP-3 for replication organelle formation, though AP-3 deficiency selectively impairs JEV and DENV RNA synthesis while inducing HPS-2 (151, 152) (**Figure 2E**). HHV-7 further exploits AP-3μ3 via its U21 protein to divert MHC-I molecules to lysosomes for immune evasion, countered by host μ3 regulatory mechanisms (110). Evolutionary divergence in viral hijacking is evident in HRSV, which engages μ3A via YXXL motifs in its matrix (M) protein to drive assembly (153), versus Nipah virus, which manipulates β3A hinge dynamics to subvert budding (154). Conversely, AP-3 also exerts antiviral functions, as SARS-CoV-2’s envelope (E) protein is sequestered by β3A binding, suppressing virion release (155). The complex’s structural plasticity, including δ/σ3 recognition of [DE]XXXL[LI] motifs (156), bridges lysosomal disorder mechanisms and therapeutic opportunities, exemplified by strategies targeting HIV-1 Gag-δ binding (147), blocking HRSV M-μ3A interactions (153), or disrupting paramyxovirus budding via β3A interference (154). These multifaceted interactions position AP-3’s modular architecture as both a critical vulnerability in viral replication cycles and a template for structure-guided antiviral design, balancing its roles as a viral accomplice and host defender.

### 2.4 Rab protein

Rab GTPases—a conserved family of small GTP-binding proteins—serve as master regulators of eukaryotic membrane trafficking, governing spatiotemporal precision in vesicular transport through GTPase-driven molecular switching (157). These proteins coordinate sequential vesicle dynamics (budding (158), directional transport (159), docking (160), and fusion (161)) via GTP/GDP cycling while integrating broader cellular processes such as secretory pathway modulation (162), clathrin-independent endocytic sorting (163), lysosomal cargo processing (164), and chemotactic signal transduction through membrane compartmentalization (165). By functioning as molecular integrators, Rab GTPases establish a regulatory nexus that links core trafficking fidelity to pleiotropic host cell functions, creating diverse mechanistic interfaces exploitable by viral pathogens (**Table 2**).

**Table 2:** Functional mapping of Rab GTPases in key viral infection stages.

Rab GTPases	Virus	Infective stage	Functional outcome
Rab 1	HSV-1	Secondary envelopment	Mediates envelope glycoprotein ERGIC shuttling (186)
Rab 1b	HCV	Assembly stage	Directs Golgi trafficking for glycoprotein maturation (182)
Rab 5	JEV	Entry stage	Coordinate clathrin-mediated endocytic sorting (167)
Rab 5c	ADV, RSV	Replication stage	Hijacks autophagy flux to amplify viral genome replication (170)
Rab 6	HSV-1	Release stage	Facilitates secretory vesicle biogenesis as an alternative release pathway (188)
	PRV	Assembly/Egress	Redirects retrograde transport to evade immune surveillance (173)
Rab 7	FMDV, ZIKV	Entry stage	Disrupts endosomal maturation pathway (168, 169)
	IAV	Immune evasion	Inhibit autophagosome-lysosome fusion (190)
	RSV	Multi-stage	Disrupts cholesterol homeostasis (14)
Rab 9	HCV	Release stage	Mediates LE-plasma membrane fusion for enveloped release (184)
	HSV-1	Entry stage	Sequential endosomal maturation for nuclear entry (187)
Rab 11	MARV	Assembly/Egress	Hijacks secretory pathway for progeny release (174)
Rab 11a	HCV	Assembly/Egress	Enhances cell-to-cell spread via recycling endosomes (183)
Rab 18	DENV	Replication stage	Utilizes membrane curvature for replication organelle formation (171)
	HCV	Assembly stage	Coordinates core protein-lipid droplet delivery (180)
	PRRSV2	Assembly/Egress	Mediates lipophagy-dependent viral particle envelopment (172)

Rab GTPases serve as indispensable host factors enabling viral proliferation through spatiotemporal regulation of vesicular trafficking across infection stages (166). During viral entry, JEV exploits Rab5/Rab11-coordinated endocytic sorting (167), while FMDV and ZIKV subvert Rab5/Rab7-mediated endosomal maturation (168, 169). Replication strategies diverge: ADV and RSV hijack Rab5c-driven autophagic flux to amplify genome synthesis (170), whereas DENV utilizes Rab18-dependent membrane curvature for replication organelle formation (171). In assembly/egress phases, PRRSV2 co-opts Rab18-governed lipophagy for virion envelopment (172), PRV redirects Rab6-controlled retrograde transport to evade immunity (173), and MARV hijacks Rab11-modulated secretory pathways for progeny release (174). Strikingly, RSV exemplifies multifunctional exploitation—disrupting Rab7–RILP (Rab-interacting lysosomal protein) complexes to impair cholesterol homeostasis while commandeering Rab11a-enriched recycling endosomes to enhance cell-to-cell spread (14, 175)—highlighting Rab networks as versatile targets for viral hijacking.

Rab GTPases emerge as universal conductors for viral exploitation of membrane trafficking systems, operating with precise spatial and temporal control throughout infection cycles (**Figure 3**). During early infection, Rab5 orchestrates clathrin-mediated endocytic sorting to facilitate viral entry (176), while Rab7 governs endosomal maturation pathways that bifurcate towards either lysosomal degradation or replication-optimized compartments (177). Post-uncoating, Rab5A stabilizes ER-associated replication complexes to amplify viral RNA synthesis (178), concurrently partnering with Rab27A to shield viral genomes within transport vesicles from cytoplasmic immune sensors (179). The viral subversion of Rab networks extends through particle assembly to egress: HCV demonstrates a multi-Rab assembly cascade where Rab18/32 coordinate core protein-lipid droplet delivery (180, 181), Rab1b directs Golgi transit for glycoprotein maturation, and Rab11a activates RE machinery for pre-assembly intermediate processing (182, 183), culminating in Rab9-TIP47-mediated LE-plasma membrane fusion for enveloped release (184) (**Figure 3D**). HSV-1 expands this manipulation by sequentially engaging Rab5/Rab7/Rab9 for nuclear entry through endosomal maturation (185), followed by Rab1-mediated ERGIC shuttling of envelope glycoproteins and Rab43/Rab9/Rab11-driven TGN sorting during secondary envelopment (186, 187). The virus further subverts Rab11 recycling pathways for non-canonical plasma membrane egress while exploiting Rab6-mediated secretory vesicle biogenesis and Rab43/Rab27A-dependent vesicle docking for alternative release routes (188, 189) (**Figure 3C**). Notably, certain viruses inversely exploit Rab inactivation for immune evasion, exemplified by IAV hijacking M2 protein to block Rab7-mediated autophagic degradation: by disrupting Rab7-TBC1D5 interaction, M2 prevents GTP hydrolysis, trapping Rab7 in an inactive state. This selective impairment of Rab7 activity aborts autophagosome-lysosome fusion, preserving intracellular virions from degradation while enabling their plasma membrane budding (190) (**Figure 1D**). These coordinated strategies – ranging from Rab activation for trafficking optimization to targeted Rab inactivation for immune escape – reveal Rab GTPases as dual-purpose nodes whose conserved yet adaptable regulatory mechanisms are systematically repurposed across viral taxa. The evolutionary convergence on Rab-centric membrane trafficking subversion underscores their position as prime therapeutic targets for intercepting viral transport logistics across infection paradigms.

## 3 Vesicular trafficking mechanisms are effective antiviral targets

**Figure 5:**
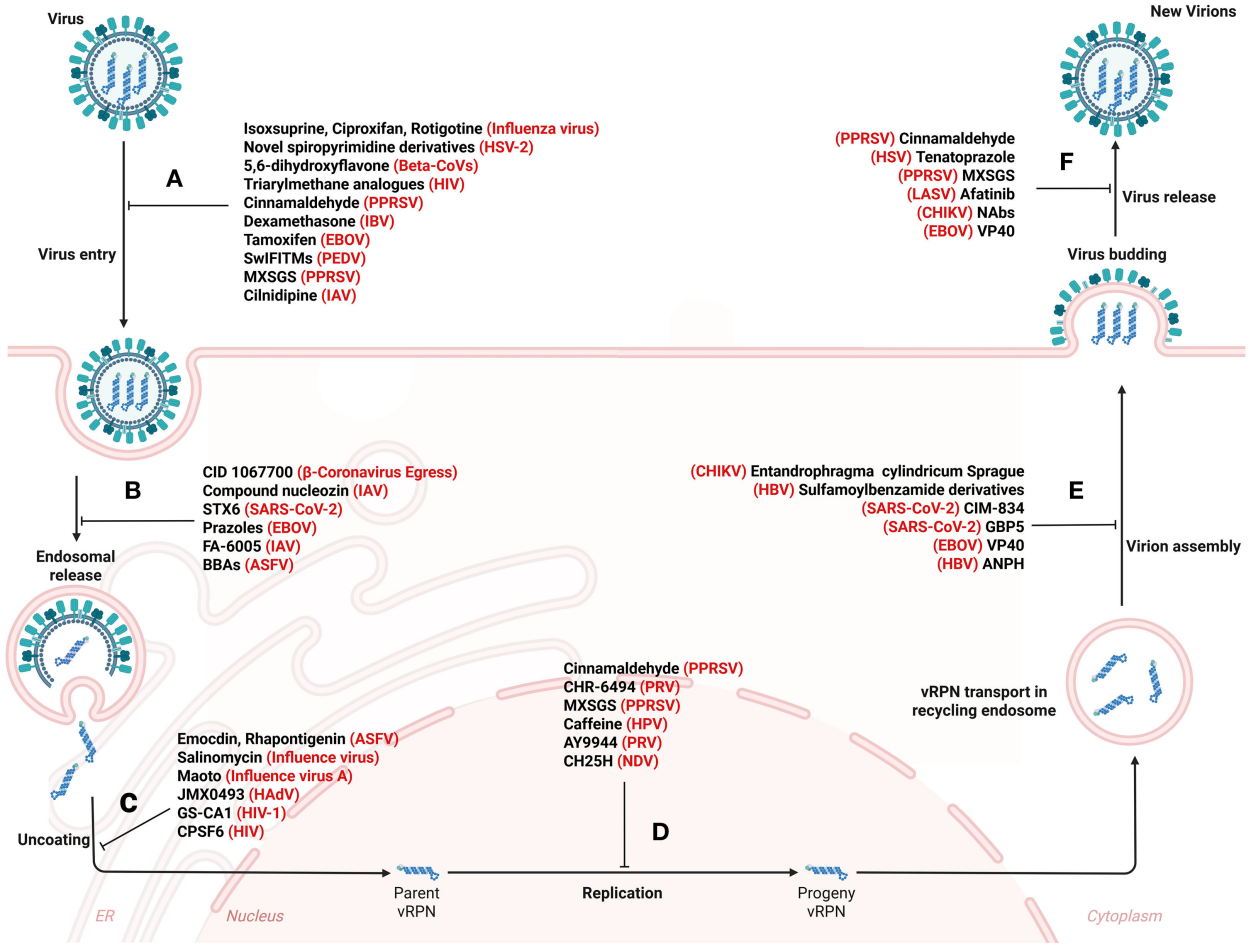
Systemic disruption of viral propagation: therapeutic roadblocks from invasion to particle release. Small molecule drugs act on vesicle transport pathways, thereby affecting virus attachment and entry **(A)**, endosomal vesicle transport **(B)**, uncoating **(C)**, replication **(D)**, assembly **(E)**, budding and release **(F)**. The detailed antiviral mechanism is shown in **Table 3**. Figure created with BioRender.

Viral infections persistently threaten global public health, driving an urgent need for innovative pharmacological strategies to counteract rapidly evolving pathogens. Small-molecule inhibitors and host-directed antivirals have emerged as key pillars of antiviral therapy, offering distinct advantages over traditional approaches. These agents disrupt viral proliferation through a dual mechanism: targeting either evolutionarily conserved host pathways—such as those regulating viral replication complexes or entry receptors [e.g., 1,3-diphenylurea derivatives inhibit viral entry (191), Tubeimosides blocking endosomal fusion (192)]—or hijacking pathogen-specific vulnerabilities (**Figure 5**) (**Table 3**). For example, the small-molecule inhibitor EDP-235 achieves broad-spectrum antiviral activity by irreversibly binding to the highly conserved 3CL protease (3CLpro), which is an essential viral enzyme for the replication of coronaviruses such as SARS-CoV-2 (193). In contrast, CD-MUS—an entry inhibitor based on β-cyclodextrin—targets the host heparan sulfate proteoglycan (HSPG), a universal entry receptor utilized by various respiratory viruses including RSV and DENV. This inhibitor blocks viruses’ attachment through multivalent sulfonate interactions and irreversibly neutralizes particles across multiple virus families (194). The dual approach leverages host-pathogen interdependence to achieve broad-spectrum efficacy against diverse RNA viruses while minimizing drug resistance development.

**Table 3:** Vesicle trafficking mechanisms can be used as promising drug targets.

Antiviral targets	Name	Virus	Antiviral mechanism/target
Protein/ enzyme inhibitor	swine interferon-induced transmembrane proteins	PEDV	confining PEDV within caveolin-1 (354)
	Cholesterol 25-hydroxylase (CH25H)	NDV	viral NP (355)
	Neutralizing antibodies (NAbs)	CHIKV	CHIKV glycoproteins (356)
	CID1067700	β-Coronavirus Egress	Rab7 GTPase (357)
	syntaxin-6	SARS-CoV-2	endocytic release (358)
	CHR-6494	PRV	Haspin (359)
	CIM-834	SARS-CoV-2	M protein (360)
	VP40	EBOV	assembly, budding, and spread (361)
	afatinib	LASV	LASV vRNP activity (362)
Small molecule inhibitors	N-(4-Nitrophenyl)-1-phenylethanone hydrazone	HBV	HBV genome (363)
	triarylmethane analogues compound L14	HIV-1	Lys35, Gln38 and Trp32 residues (364)
	G protein-coupled receptor antagonists	EBOV	Replication (365)
	NSF attachment protein inhibitors	SARS-CoV-2	Transport (248)
	prazole proton pump inhibitor	EBOV	ESCRT-1 factor Tsg101 (366)
	protein kinase D inhibitors	SARS-CoV-2	Replication (367)
	cyclosporine derivatives	HBV	Entry (368)
	Pitstop 2c、Pitstop 2d	VSV	Endocytosis (369)
	AAK1 inhibitors	SARS-CoV-2	Entry (370)
	tetrandrine	EBOV	Transport (221)
	JMX0493	HAdV	Inhibition of endosomal escape (371)
	maraviroc	HIV	Entry (372)
	FA-6005	IAV	viral ribonucleoproteins (373)
	Caffeine	HPV	Replication (374)
Drugs/Compounds	novel spiropyrimidine derivatives compound 3	HSV-2	caspase-3 protein expression (375)
	Emodin (EM) and rhapontigenin (RHAG)	ASFV	Rab 7 protein expression level (376)
	Entandrophragma cylindricum Sprague	CHIKV	Entry and replication (377)
	isoxsuprine, ciproxifan, rotigotine	Influenza virus	neurotransmitter receptors (378)
	5,6-dihydroxyflavone (5,6-DHF)	Beta-CoVs	receptor binding domain (RBD) of spike (379)
	bis-benzylisoquinoline alkaloids	ASFV	endosomal/lysosomal (380)
	sulfamoylbenzamide derivatives	HBV	HBV capsid (381)
	chebulagic acid and punicalagin	RSV	Attachment and membrane fusion (382)
	ilaprazole, tenatoprazole	HSV	TSG 101 (383)
	Ma-Xing-Shi-Gan-San	PRRSV	ALB, PPARG, CASP3, STAT3, etc (384)
	amiodarone analogues	EBOV	Fusion (385)
	cinnamaldehyde (CA)	PPRSV	PRRSV N protein (386)
	dexamethasone (Dex)	IBV	GR and NHE3 (387)
	cepharanthine	ASFV	Attachment and internalization (388)
	salinomycin	Influenza virus	viral matrix protein 2 (389)
	cilnidipine	IAV	HA2 subunit (390)
	tamoxifen	EBOV	EBOV-glycoprotein (GP) (391)
	nucleozin	IAV	viral nucleoprotein (392)
	AY9944	PRV	7-dehydrocholesterol reductase (393)
	GS-CA1	HIV-1	HIV-1 capsid (394)
	suramin	CHIKV	Entry (395)
	matrine	PEDV	Attachment and entry (396)
	maoto	IAV	vacuolar-type H ATPase (397)
	Gly-9	Prion	Reduce protein levels (398)
miRNA	miR-221、miR-222	HIV-1	Entry (399)
	miR-155-5p	DENV	Genes that regulate interactions (209)
	miR-142-3p	Uukuniemi	Intracellular transport (400)
	miR-185	SARS-CoV-2	Entry (401)
	miR-204	PRRSV	Inhibition of autophagy (402)
others	cleavage and polyadenylation specificity factor 6	HIV-1	HIV-1 capsid (403)
	ISG guanylate-binding protein 5 (GBP5)	SARS-CoV-2, HIV	N-linked glycosylation (404)
	IFITM family of restriction factors	ZIKV	Viral infection (405)
	interferon stimulated genes	DENV	Transport (406)
	BST-2/Tetherin	HIV-1	Release (407)

### 3.1 Orchestrating vesicular hijacking: multimodal antiviral strategies targeting the host pathogen interface

Antiviral therapies achieve multimodal disruption of viral life cycles through two complementary mechanisms—vesicle/organelle manipulation and host pathway engineering—that synergistically intercept vesicular trafficking networks. Specifically, vesicle rerouting strategies exploit transport pathway vulnerabilities to generate non-infectious virions, as exemplified by Rab5b-mediated rerouting diverting HBV surface proteins from ER trafficking to multivesicular body (MVB) sequestration (195), This spatial interference simultaneously activates MAVS-dependent mitochondrial antiviral signaling while exposing viral particles to innate immune “double hits” through enhanced type I interferon secretion prior to degradation (196). Similarly, Copb2 depletion traps SARS-CoV-2 progeny within ERGIC via COPII vesicle blockade (197), a mechanism that paradoxically improves MHC-I antigen presentation efficiency as proteasomes more effectively process viral antigens retained in ERGIC compartments (198). By exploiting viral evolutionary dependence on host trafficking systems, these tactics limit viral adaptation to therapeutic pressure while preserving cellular homeostasis. Complementing spatial diversion approaches, membrane fusion sabotage mechanisms are typified by interferon-induced transmembrane protein 3 (IFITM3), which neutralizes PRRSV fusion capacity through cholesterol-driven membrane curvature generation, creating steric hindrance for envelope fusion and reducing progeny infectivity (199). Notably, IFITM3 stabilizers under clinical evaluation demonstrate synergistic effects when combined with endocytosis inhibitors, though their lipid-modifying activity demands rigorous lipidomic monitoring to mitigate off-target effects on cholesterol metabolism in non-infected cells (200).

The latest research demonstrates that the dengue virus nonstructural protein achieves immune escape through a multistep mechanism involving PRDM1-mediated downregulation of host Nod-like receptor (NLRP12), hijacking of heat shock protein 90 (HSP90), and disruption of the NLRP12-HSP90 interaction essential for type I interferon signaling. This coordinated suppression not only inhibits antiviral interferon-stimulated genes such as IFITM3 but also cripples host defenses by dismantling the interferon pathway. These findings support therapeutic strategies combining IFITM3 stabilizers with endocytosis inhibitors to counteract viral immune evasion (201). Emerging clinical paradigms further integrate these approaches with PD-1 blocking antibodies, leveraging the dual benefit of viral replication suppression (via trafficking disruption) and T-cell reinvigoration (through checkpoint inhibition)—a strategy currently validated in preclinical models of chronic HBV coinfection (202). Concurrent interventions leverage interferon lambda 1 (IFN-λ1) to enhance autophagic flux through ATG10 activation, synchronizing accelerated lysosomal degradation (42) with reprogramming of intracellular trafficking networks via Rab1-regulated ER-Golgi transport, Rab11-dependent recycling endosome dynamics, and Rab6-mediated secretory vesicle exocytosis. The strategy further incorporates STING agonists to amplify the cGAS pathway activation, enabling autophagy-encapsulated viral DNA to trigger secondary interferon waves and establish a self-reinforcing innate immunity loop. This multilayer approach mirrors natural host defenses by converging modular viral lifecycle interventions with systemic cellular rewiring to undermine pathogen adaptability. Through simultaneous disruption of host-pathogen coadaptation and amplification of innate antiviral mechanisms, the dual-axis framework provides a blueprint for broad-spectrum therapies that target viral plasticity while maintaining cellular homeostasis.

Enzyme-targeted antiviral strategies achieve multimodal disruption of viral trafficking through three spatially coordinated mechanisms. The first axis targets ADP-ribosylation factor (ARF) GTPases, master regulators of post-Golgi transport. Their inhibition traps ZIKV, IAV, and SARS-CoV-2 virions in compromised Golgi compartments, forcing lysosomal degradation through impaired export competence (203). This leverages viruses’ evolutionary dependence on conserved host trafficking machinery, which constrains their mutational escape from such interventions. The second mechanism involves PIKfyve kinase blockade, which induces rapid phosphoinositide depletion to collapse ESCRT-dependent endosomal maturation. This dual-action strategy dismantles replication niches in VSV and ZIKV infections while suppressing NF-κB-driven inflammation (204). Concurrently, the resulting mitochondrial DNA stress activates the cGAS-STING pathway, establishing an interferon-independent antiviral state that broadens protection against diverse RNA viruses (205). The third frontier focuses on sphingosine kinases SK1/SK2. Their inhibition executes biphasic containment: chemically, it inverts ceramide-to-S1P ratios to disrupt LE-lysosome fusion in coronaviruses and EBOV; mechanically, it induces cortical actin polymerization to stiffen endocytic membranes, physically blocking enveloped virion penetration (206). This therapeutic triad—intercepting vesicle maturation checkpoints (ARF GTPases), destabilizing replication organelle integrity (PIKfyve), and reinforcing membrane rigidity (SK1/SK2)—establishes complementary metabolic barriers that counteract evolutionarily conserved viral entry/egress mechanisms through spatial compartmentalization. By strategically mirroring the layered defense logic of innate immunity, this tripartite strategy effectively converts the host’s subcellular compartmentalization into an insurmountable fortress against viral invasion through coordinated structural and functional interventions. Notably, current preclinical research has begun exploring the combination of this enzyme-targeting strategy with PD-1 blocking antibodies, which concurrently achieves viral replication suppression and cellular exhaustion alleviation (207). This dual therapeutic effect thereby demonstrates significant potential for transforming conventional antiviral approaches into an integrated paradigm of immune reconstitution therapy, as evidenced by recent experimental findings.

### 3.2 Vesicle-mediated antiviral drug delivery and miRNA modulation

The intricate interplay between viral pathogenesis and host vesicular transport systems has catalyzed therapeutic innovation through mechanistic insights, thereby driving the development of vesicle-centric strategies to intercept viral life cycles. Notably, vesicle-mediated drug delivery systems are emerging as precision tools targeting intracellular trafficking nodes, exemplified by the nitazoxanide derivative tizoxanide, which suppresses DENV-2 replication through dual disruption of vesicular transport dynamics and host post-translational modification pathways (208). These interventions exploit the evolutionary persistence of core cellular logistics networks, imposing mutational ceilings on viral escape by anchoring therapeutic pressure to immutable host constraints. Concurrently, microRNA-155-5p (miR-155-5p) orchestrates a biphasic antiviral response that simultaneously downregulates interferon signaling, TP53 tumor suppressor pathways, and vesicle trafficking machinery while potentiating cellular defense mechanisms, revealing novel therapeutic targets in dengue pathogenesis (209). This layered regulation exemplifies a systems-level therapeutic paradigm where host network modulation at critical inflection points supersedes direct viral targeting. Intriguingly, the ER-anchored STING protein exhibits paradoxical functionality: it inhibits coronavirus replication by blocking ER-derived double-membrane vesicle biogenesis (210), yet acts as a proviral scaffold for RNA viruses such as enterovirus D68 and SARS-CoV-2 during replication complex assembly (211). Such duality underscores the inherent biological trade-offs in manipulating host machinery—balancing antiviral potency against the risk of inadvertently supporting viral adaptation. Collectively, these discoveries delineate vesicle-centric therapeutic landscapes while emphasizing their translational potential and context-dependent biological constraints.

Extracellular vesicle (EV)-transported miRNAs coordinate antiviral defense by dually regulating viral replication dynamics and host immunomodulatory networks. Mechanistically, miR-431-5p establishes antiviral states by silencing CD95-mediated apoptotic signaling (212), while miR-136-5p suppresses M1 macrophage polarization through simultaneous inhibition of GNAS/PI3K/ERK/STAT3 signaling pathways, thereby attenuating viral-induced hepatic inflammation (213). This regulatory network reveals a new dimension in HBV infection: IFNα-activated macrophages deliver interferon-stimulated gene (ISG) products to HBV-infected hepatocytes via exosome secretion, significantly inhibiting HBV replication. Notably, the aldehyde glyoxylate system (AGS) enhances HBV replication while suppressing ISG activation by hijacking the host vesicle trafficking pathway. The exosome release inhibitor GW4869 completely abrogates IFNα’s antiviral effects, confirming exosomes as the central mediators of macrophage-hepatocyte antiviral crosstalk (214). This discovery uncovers a new paradigm of vesicle-mediated immune regulation, whereby activated immune cells restore ISG functionality in virus-infected cells through exosomal signaling, providing a host-directed precision strategy for chronic hepatitis therapy. This immune homeostasis modulation reflects an evolutionary balance between pathogen eradication and tissue protection, encoded within EV-mediated miRNA circuits through pathogen-host coadaptation. Additionally, EV-encapsulated miRNA clusters (miR-15b-5p, miR-24-3p, miR-223-3p) enhance mRNA vaccine efficacy by boosting antigen-presenting cell activation and germinal center B-cell responses (215), contrasting with milk-derived miR-let-7e and miR-27b that directly inhibit PEDV RNA-dependent RNA polymerase (RdRp) to block replication (216). Notably, mesenchymal stem cell-derived EVs enriched with the miR-92a-3p/26a-5p/23a-3p triad exert dual antiviral effects—suppressing SARS-CoV-2 genomic RNA synthesis via NSP12 helicase interference while mitigating NF-κB/IL-6-mediated cytokine storms in infected alveolar epithelium (217). This multidimensional strategy exploits the evolutionary conservation of viral replication checkpoints, restricting viral adaptability and co-opting host stress-response pathways to establish antiviral barriers. Together, these interconnected regulatory mechanisms highlight the therapeutic potential of EV-miRNA engineering for precision targeting of pathogen-host interactions across divergent viral families.

### 3.3 Small molecule inhibitors targeting host vesicular checkpoints against viral escape

Host-directed antiviral therapeutics overcome viral drug resistance by targeting evolutionarily conserved cellular machinery that governs pH homeostasis and vesicular trafficking, thereby establishing complementary defense layers against viral adaptation through three interdependent mechanisms: neutralizing endosomal acidification to block viral fusion, disrupting vesicular transport dynamics to prevent capsid uncoating, and competitively saturating host receptors to inhibit viral attachment. This strategy capitalizes on the host pathways’ evolutionary constraints, which viruses cannot readily mutate to bypass, thus creating durable antiviral barriers shaped by co-evolutionary pressures. Pharmacologically, pH regulation inhibitors like obatoclax exemplify this approach by inducing endosomal alkalinization, which locks coronavirus spike glycoproteins in prefusion conformations and renders virions fusion-incompetent (218). Notably, obatoclax demonstrates potent antiviral activity against SARS-CoV-2 in TMPRSS2-negative cells (EC_50_ = 0.06 μM) through pH-dependent entry blockade, with achievable therapeutic plasma concentrations (~0.4 μM) via intravenous administration. While phase I/II oncology trials established its safety profile at 20–40 mg/m² doses, transient neurological symptoms (grade 1-2) and reversible hepatotoxicity remain dose-limiting considerations for antiviral repurposing (219). Similarly, chloroquine and bafilomycin A1 disrupt acidification-dependent penetration routes, as confirmed by cryo-electron tomography, achieving comparable entry blockade through spatial interference (220). Beyond pH modulation, vesicular trafficking inhibitors such as tetrandrine suppress SARS-CoV-2 via dual mechanisms: impairing endolysosomal maturation through calcium flux disruption mediated by two-pore channel 2 (TPC2) while paradoxically stabilizing viral ribonucleoprotein complexes (221). Recent studies highlight tetrandrine demonstrates broad experimental efficacy, with reversible hepatosteatosis as a key toxicity linked to oral route-induced portal venous accumulation. Pulmonary delivery or structural analogs may mitigate hepatic risks while preserving antiviral activity, particularly in short-term SARS-CoV-2 regimens (222). These interventions are further amplified through synergistic therapeutic architectures, where calpeptin enhances remdesivir’s antiviral activity by simultaneously inhibiting papain-like protease-mediated polyprotein cleavage and host caspase-8-dependent viral egress (223). Such combinatorial strategies exploit viral dependency networks that bridge host-pathogen interfaces, effectively turning cellular defense mechanisms against viral replication checkpoints. For receptor competition, spatial hindrance is achieved through MDM2 antagonists like nutlin-3a, which mobilize ACE2-enriched EVs to saturate SARS-CoV-2 binding interfaces (224). Intriguingly, even replication fidelity mechanisms prove targetable, as camptothecin derivatives terminate flavivirus RNA synthesis through topoisomerase 1-DNA cleavage complex stabilization, effectively stalling replication (225). By synchronously intercepting viral lifecycle checkpoints while preserving host proteostatic resilience, this systems-level approach constructs an evolutionary firewall against pathogen adaptability, thereby providing a blueprint for preemptive countermeasures against viral mutational escape.

## 4 Vesicle small molecules serve as detection molecules for viral infection status

The co-option of host-derived EVs by viruses exemplifies an evolutionary refinement strategy that facilitates both stealth dissemination and systemic manipulation of cellular communication networks. Specifically, through pathogen-encoded molecular machinery, viruses subvert host proteostasis systems and commandeer vesicular trafficking pathways to reconfigure EV biology. This hijacking is epitomized by SARS-CoV-2, HIV-1, and HTLV-1, which remodel EV biogenesis through three-dimensional modulation of miRNA cargo profiles, immune-evasive surface marker expression, and vesicular dimensional plasticity to establish systemic infection (226–230). Hubert et al. (231) combined nanoparticle tracking analysis and enzymatic activity mapping to reveal an inverse correlation between circulating EV abundance and host miRNA bioavailability. Their study further demonstrated that reduced EV diameters quantitatively correlated with lymphocyte activation thresholds. These findings establish EV-mediated miRNA trafficking as a critical regulator of viral latency states (232). Such systemic rewiring underscores the dual-edged nature of EVs: their ancient role in cellular crosstalk becomes both a weapon and a vulnerability in host defense architectures. These findings not only position quantitative EV subpopulation profiling as a transformative liquid biopsy modality for monitoring infection progression and therapeutic efficacy through vesicle-encoded molecular fingerprints but also highlight EVs’ dual role as multimodal signaling platforms. Functioning as composite molecular carriers, EVs transport non-coding RNAs, post-translationally modified proteins, and bioactive lipids while executing precision regulation of intercellular crosstalk via spatiotemporally controlled signal transduction. Such dynamic orchestration coordinates pan-tissue homeostasis and immune microenvironment remodeling, ultimately rendering EVs pivotal yet vulnerable nodes in the host-pathogen interface.

As a key molecule of gene regulation, miRNAs carried by vesicles have shown important value in disease dynamic monitoring and precise diagnosis and treatment by targeting and regulating mRNA expression. First, exosomal miRNAs in HCV-infected patients function as dynamic biomarkers for monitoring host-virus interactions and antiviral immune response kinetics (233), whereas integrative profiling of SARS-CoV-2 Spike S1-bearing EVs against innate and adaptive immunity-associated EV signatures enables tissue-resolved tracking of viral reservoir persistence and disease severity stratification (234). These vesicular biomarkers exploit the evolutionary logic of intercellular communication systems, where viruses inadvertently co-opt host-derived signaling architectures to disseminate but in doing so create diagnostic vulnerabilities through molecular footprint leakage. Based on this, these technological advancements establish vesicular biomarkers as multidimensional diagnostic platforms capable of three-dimensional mapping: viral pathogenesis trajectories through longitudinal cargo analysis, therapeutic target engagement via pharmacodynamic marker quantification, and clinical outcome prediction using machine learning-driven signature deconvolution. Further, the molecular characteristics based on EV have demonstrated diagnostic precision across heterogeneous pathological contexts, including infection dynamics (Chagas disease parasite load monitoring (235), baculovirus replication kinetics (236), HIV-1 disease progression staging (237)), oncolytic virotherapy response assessment (immune activation states in adenovirus-treated tumors (238)), and neoplastic management (nasopharyngeal carcinoma immunotherapy efficacy (239, 240), glioblastoma tumor microenvironment profiling (241), metastatic colorectal cancer liquid biopsy (242)). The universality of EV-mediated diagnostics arises from their capacity to integrate multimodal cellular stress signatures—capturing both the immediate perturbations of pathogens and the adaptive reprogramming of host survival networks—thus positioning vesicles as sentinels of system-wide pathophysiological equilibrium shifts. Notably, vesicular biomarkers demonstrate cross-species applicability in simian immunodeficiency virus pathogenesis modeling (243) and veterinary diagnostics, particularly for porcine hemagglutinating encephalomyelitis virus surveillance (244). Their clinical utility further extends to specialized scenarios, including therapeutic resistance prediction in castration-resistant prostate cancer (245), severity indexing for pediatric viral pneumonia (246), and treatment response monitoring in tuberculosis (247). In addition, the emerging applications in hematologic malignancies (acute myeloid leukemia minimal residual disease detection (248)) and developmental disorders (nephroblastoma differential diagnosis (249)) further validate their pan-pathological diagnostic versatility.

## 5 Conclusion

Viruses, as obligate intracellular parasites, critically depend on host vesicular trafficking systems to execute their life cycles. Specifically, these pathogens systematically exploit membrane transport machinery to coordinate successive infection stages, including viral entry via receptor-mediated endocytosis (250), envelope fusion with host membranes (251), replication complex formation for genome amplification (252), virion assembly at specialized organelle interfaces (253), budding through membrane scission mechanisms (254), and extracellular release via exocytic pathways (255). While these trafficking networks are essential for cellular homeostasis, their functional complexity paradoxically constitutes an exploitable vulnerability for viral pathogenesis. Evolutionarily, viruses optimize strategies to hijack transport pathways for establishing replication niches, thereby underscoring the necessity to decode virus-host interplay at membrane trafficking checkpoints. For instance, elucidating molecular blueprints through which divergent viral families co-opt cellular conduits—from repurposing ER-Golgi intermediate compartments to retroviruses hijacking multivesicular bodies—not only provides dual benefits (advancing mechanistic virology and illuminating druggable targets) but also mandates the development of precision therapeutics. Such therapeutics must selectively inhibit viral glycoprotein interactions with vesicle-associated adaptors while sparing physiological transport functions—a strategy requiring combinatorial approaches integrating structural virology and membrane proteomics.
